# Supplementary material for: TERMINAL FLOWER 1-FD complex target genes and competition with FLOWERING LOCUS T
Source: Nat Commun. 2020 Oct 12;11:5118. doi: 10.1038/s41467-020-18782-1 (PMC7550357; doi:10.1038/s41467-020-18782-1)
Supplement: Supplementary file 1 — Supplementary Information [file 41467_2020_18782_MOESM1_ESM.docx]

**Supplementary figures:**

**
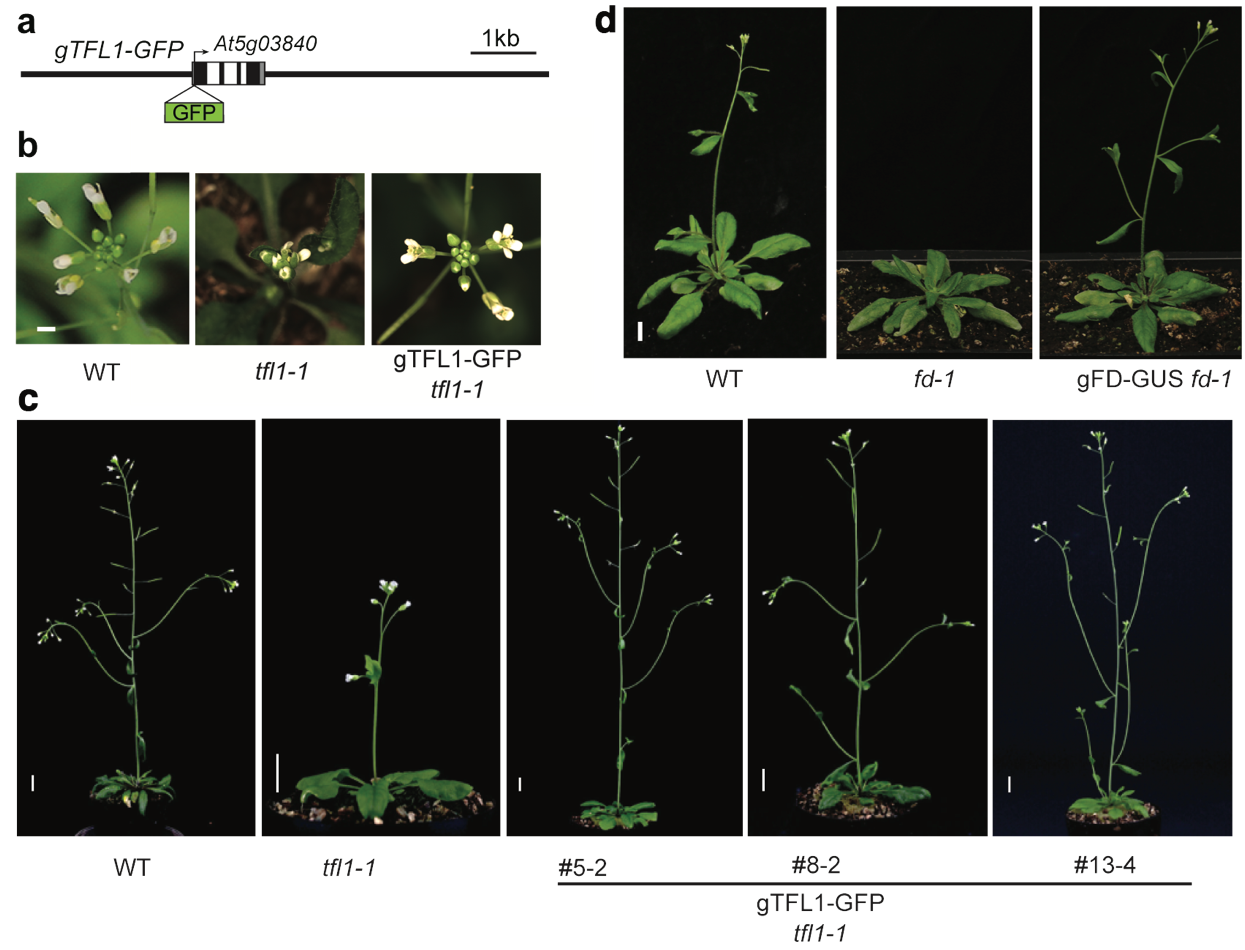
**

**Supplementary Figure 1. Biological activity of gTFL1-GFP and gFD-GUS.**

**a,** gTFL1-GFP construct. **b, c,** Rescue of the terminal flower phenotype of the severe *tfl1-1* mutant by gTFL1-GFP. Top view showing terminal flower phenotype (b). Scale bar, 5 mm. Independent transgenic lines (c). Scale bar, 1 cm. **d**, Rescue of the late-flowering phenotype of the null *fd-1* mutant by gFD-GUS ^1^. Scale bar, 1cm.

**
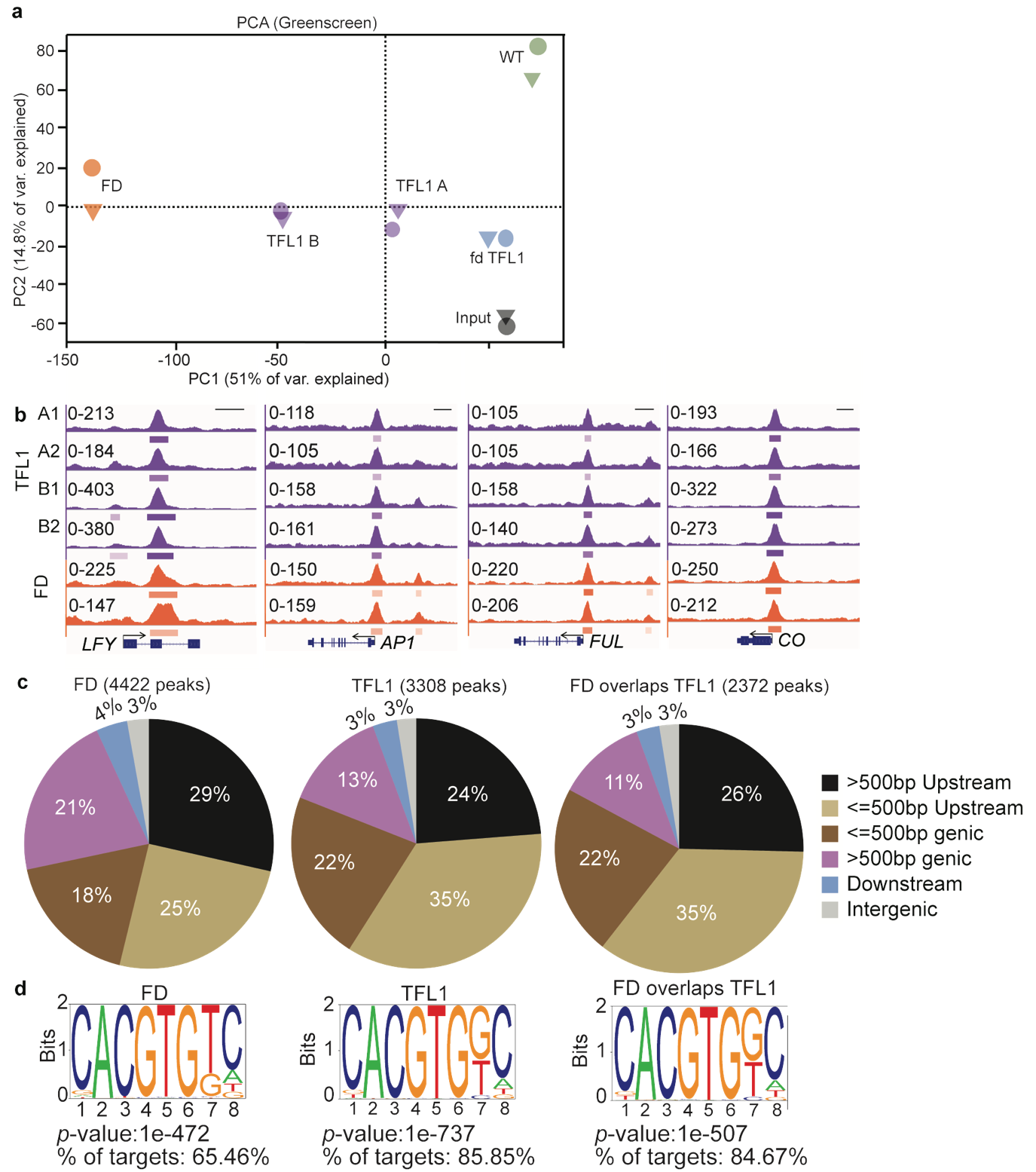
**

**Supplementary Figure 2. Distribution and *cis* motifs of significant TFL1, FD and co-bound peaks.**

**a**, Principal component analysis (PCA) of CP10M normalized ChIP-seq reads from anti-GFP ChIP in gTFL1-GFP (TFL1 A and B), *fd-1* gTFL1-GFP (*fd* TFL1), and wild type (WT; negative control), anti-GUS ChIP-seq reads for gFD-GUS (FD), and input. **b**, Browser view of peaks in individual ChIP replicates for TFL1 (A1, A2, B1 and B2) and FD. Significant peaks (summit MACS2 q value ≤10^-10^) are marked by horizontal bars, with the colour saturation proportional to the -log 10 q value (as for the narrowPeak file format in ENCODE). **c**, ChIP-seq peak summit distribution of FD, TFL1 and the overlapping peaks (TFL1 and FD) in genic and intergenic regions. Intergenic peaks are > 4kb from the closest gene. **d**, *cis* motifs most significantly enriched under TFL1 or FD peaks identified by *de novo* motif analysis (Homer ^2^). Sequence logos of position-specific scoring matrices (PSSMs) (top). Motif enrichment p-values and frequency (bottom).


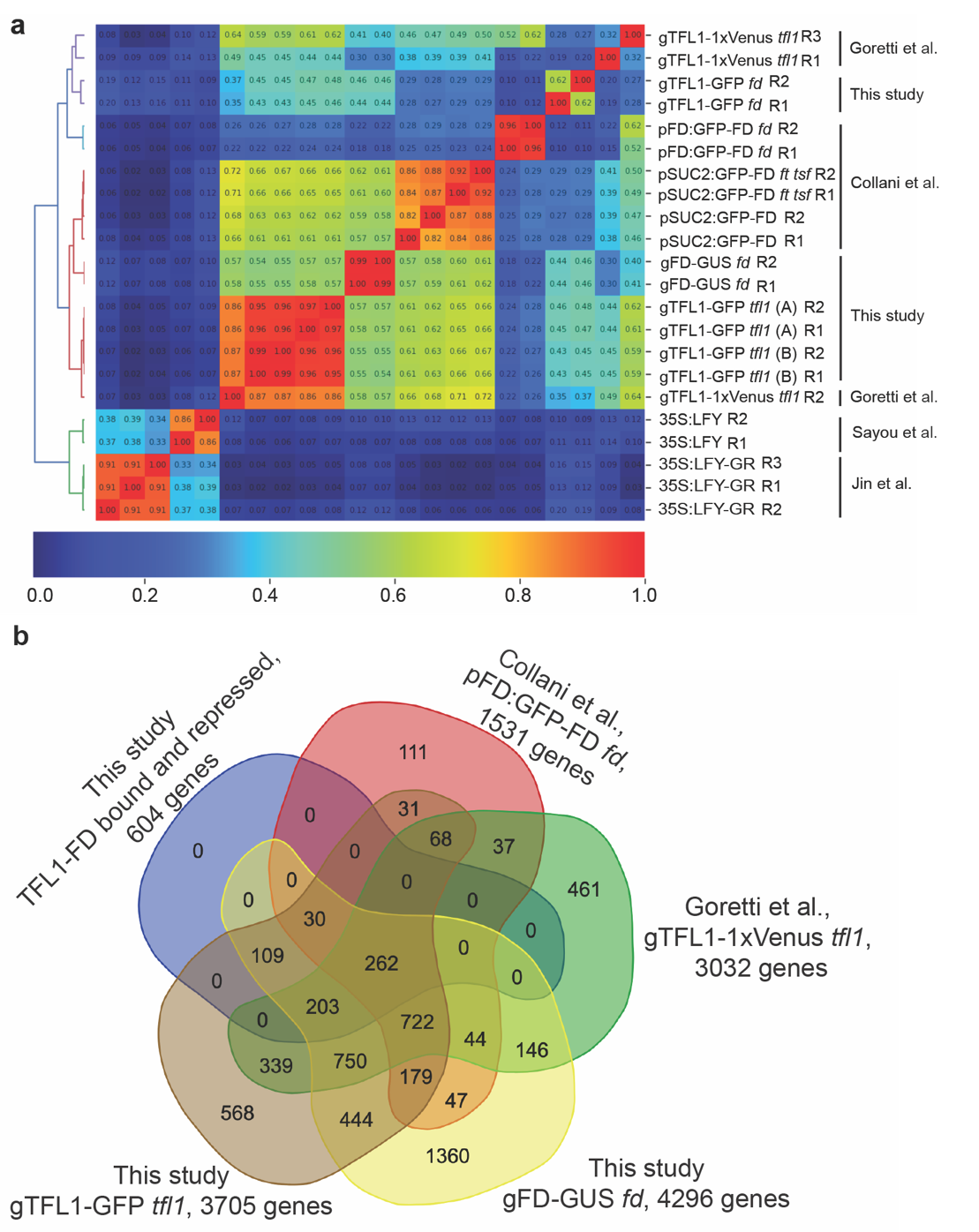


**Supplementary Figure 3. ChIP-seq quality control.**

**a**, Heatmap of Pearson correlation coefficient analysis of normalized reads of all replicates in significant peak regions (MACS2 q value ≤10^-10^). Below: legend for correlation coefficients. See Methods for details. TFL1 and FD datasets generated in the current study (42-day-old short-day-grown plants; this study) clustered together. They also clustered with recently published ChIP-seq datasets, in particular pSUC2:GFP-FD ^3^ (16 day-old long-day grown plants; Collani) and some replicates of gTFL1-1xVenus *tfl1* ^4^ (15-day-old long day grown plants; Goretti). A second, related, cluster contained pFD:GFP-FD *fd* ^3^ (16 day-old long-day grown plants; Collani), gTFL1-1xVenus *tfl1* ^4^ (15-day-old long day grown plants; Goretti) and our gTFL1 ChIP-seq in *fd* mutants. A LFY ChIP-seq experiment from our lab (root explants, 35S:LFY-GR) ^5^, together with a published LFY ChIP-seq experiment (inflorescences, 35S:LFY) ^6^, formed a separate cluster suggesting no strong batch effects. **b,** After peak calling and peak to gene annotation in analogous manner in all four ChIP-seq datasets (see Methods), we identified strong overlap between long-day and short-day endogenous TFL1 or FD peak associated genes. In addition, the long-day peak associated genes (Collani, Goretti) ^3,4^ also overlapped strongly with the 604 TFL1-FD bound and repressed genes we identified (see also Fig. 5a).


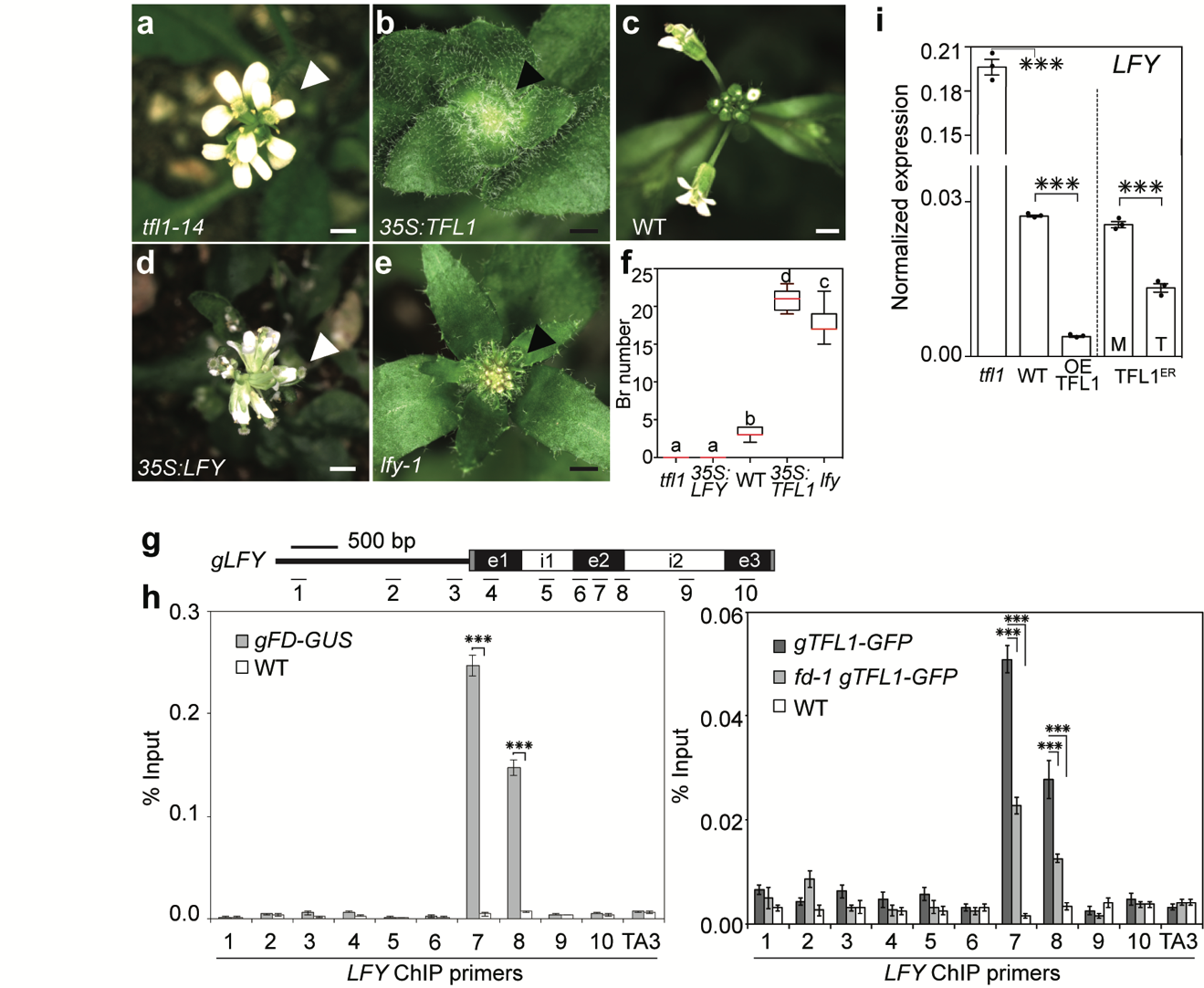


**Supplementary Figure 4. Direct repression of *LFY* by TFL1.**

**a** - **f**, Effect of LFY and TFL1 loss- and gain of function mutants on onset of flower formation in the inflorescence. Representative pictures of inflorescences (top view) grown in long-day photoperiod (a - e). Quantification of the number of branches (Br) formed (f). Scale bars: 2 mm (white) or 5 mm (black). White arrowhead: terminal flower. Box plot: median (red line; n = 12 plants), upper and lower quartiles (box edges), and minima and maxima (whiskers). Letters above boxes indicate significantly different groups; *p*-value < 0.05 based on Kruskal-Wallis test with Dunn's *post hoc* test. **g**, Diagram of *LFY* locus and ChIP primers used. **h,** Anti-GUS (left) or anti-GFP (right) ChIP of FD binding (left) and TFL1 binding (right), respectively, to the *LFY* locus in 42-day-old short-day-grown shoot apices. TFL1 binding was assayed in the presence and absence of *FD* (*fd-1* null mutant; bottom). The following controls were employed: genomic control: anti-GUS (left) or anti-GFP (right) ChIP of non-transgenic wild type (WT), internal control: occupancy at the TA3 retrotransposon locus. Shown are mean ± SEM of three independent biological experiments (black dots). P-values unpaired one-tailed *t*-test., gFD-GUS vs WT: *** region 7 = 0.0005, *** region 8 = 1E-05; gTFL1-GFP vs fd gTFL1-GFP: *** region 7 = 6E-05, *** region 8 = 0.0006; gTFL1-GFP vs WT: *** region 7 = 0.0002, *** region 8 = 2E-05. **i**, *LFY* expression in 16-day-old long-day-grown *TFL1* loss- and gain-of function mutants (left) and 4 hours after mock (M) or estradiol (T) induction of TFL1^ER^ (right). Shown are mean ± SEM of three independent experiments (black dots). *p*-values, unpaired one-tailed *t*-test: *** *tfl1*-vs-WT = 0.0004, *** WT-vs-*OETFL1* = 5E-08, *** M-vs-T = 0.0001; OE:35S:TFL1; Expression was normalized over *UBQ10*.


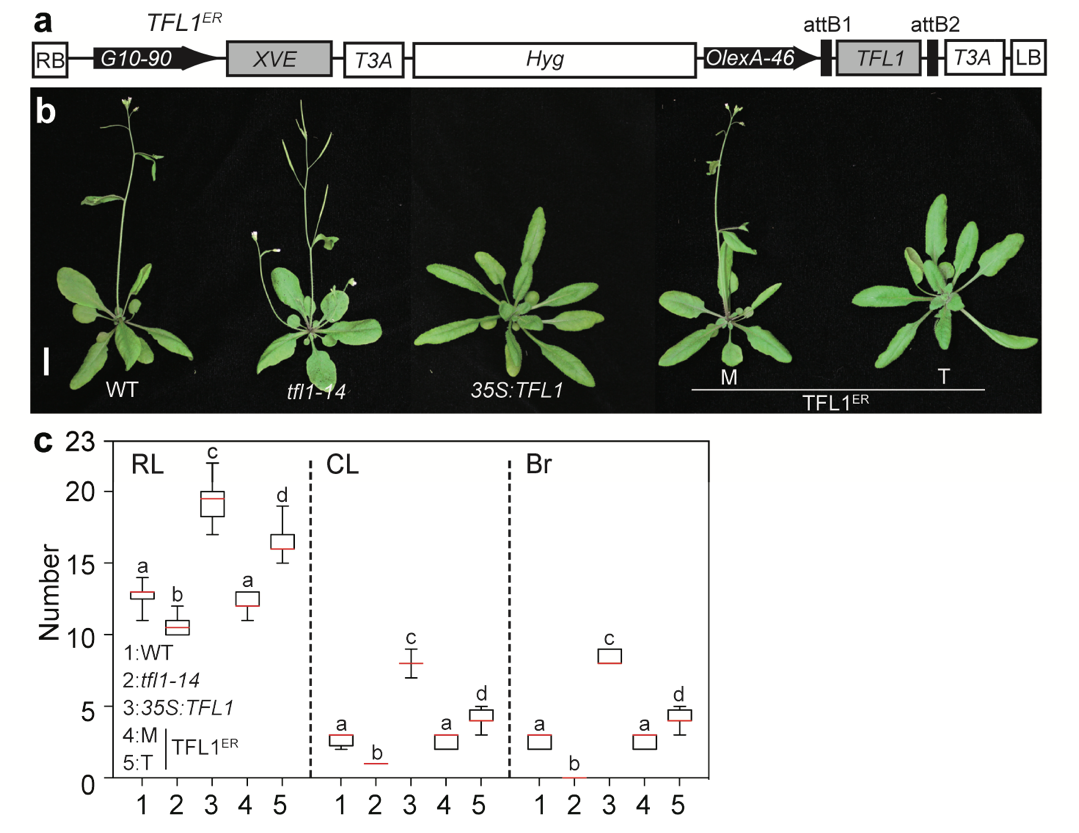


**Supplementary Figure 5. Steroid activatable TFL1 overexpression delays onset of reproductive development and flower formation.**

**a**, Construction of estradiol inducible TFL1 (TFL1 ^ER^) using pMDC7 (Ref. ^7^). Estradiol application activates the synthetic transcription factor XVE expressed from a constitutive promoter (G10-90). XVE consists of a LexA DNA binding domain (X), a VP16 activation domain (V) and an estradiol receptor hormone binding domain (E). Steroid activated XVE binds to OlexA-46 (eight copies of the *LexA* operator) in front of *TFL1* and activates gene expression. T3A: *rbcsS3A* poly(A) sequence. RB: right border, LB: left border, Hyg: hygromycin B resistance gene. **b**, Phenotypes of long-day-grown plants with decreased (*tfl1-14*), constitutively increased (35S:TFL1) or inducibly increased (TFL1^ER^) TFL1 activity. TFL1^ER^ plants were treated with 10 μmol beta-estradiol (T) or mock (M) solution from day 5 onward every other day until bolting. **c**, Quantification of phenotypes in (b). RL: rosette leaf number, CL: cauline leaf number, Br: branch number on the main inflorescence. Box plot-median (red line; n = 15 plants), upper and lower quartiles (box edges), and minima and maxima (whiskers). Letters above boxes indicate significantly different groups based on *p*-value < 0.05 Kruskal-Wallis test with Dunn's *post hoc* test.


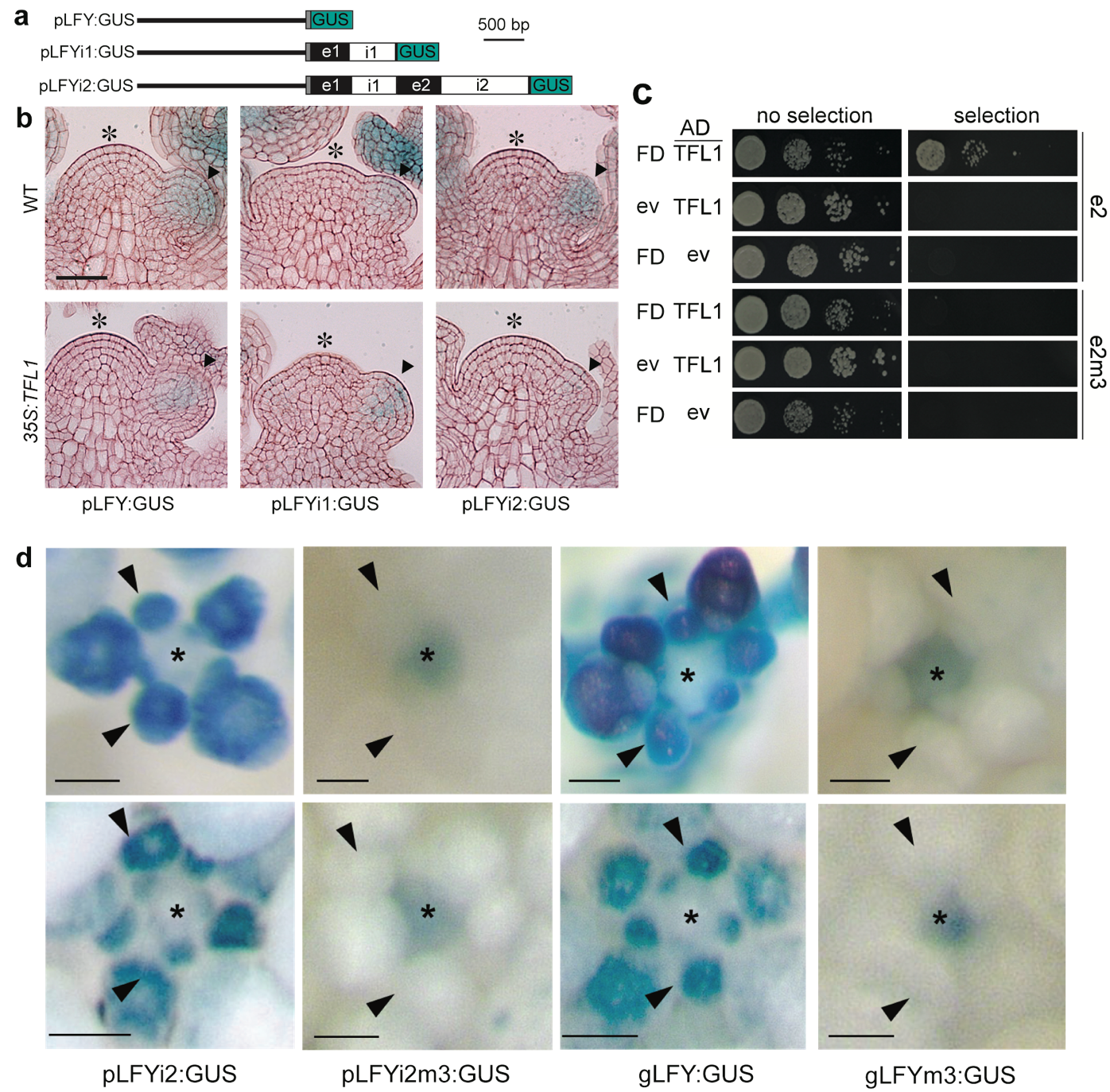


**Supplementary Figure 6. *LFY* locus regions required for repression by and recruitment of TFL1.**

**a**, LFY-GUS reporter constructs employed. Black line: 2.3 kb upstream intergenic region (*LFY* ‘promoter’) ^8^. Grey box: 5’UTR. Black boxes: exons. White boxes: introns. Blue box: beta-glucuronidase (GUS). **b**, GUS staining in wild-type (WT) and in *35S:TFL1* inflorescences grown in long-day photoperiod. Asterisk indicates the centre of the inflorescence shoot apex. Arrowheads point to flower primordia, where *LFY* is expressed. Only the pLFYi2:GUS reporter is silenced by 35S:TFL1. Scale bar: 1 µm. **c**, Test for TFL1/FD binding to wild-type (e2) and bZIP binding site mutated LFY exon 2 (e2m3) in yeast. For details on the *cis* motif mutations see Fig. 2a. LFY exon 2 or e2m3 driving expression of a resistance marker were integrated into the yeast genome. The resulting strains were transformed with plasmids expressing FD and TFL1-AD or control (ev= empty vector) and TFL1-AD. AD: activation domain. Yeast 14-3-3 proteins mediate FD-florigen interactions in yeast ^9,10^. Growth was assayed on media without or with selection. **d**, Accumulation of beta-glucuronidase in independent pLFYi2:GUS, pLFYi2m3:GUS, gLFY:GUS and gLFYm3:GUS lines. Staining was conducted under identical conditions. Arrowheads: flower primordia; asterisk: inflorescence shoot apex; Scale bars: 2 mm.

**
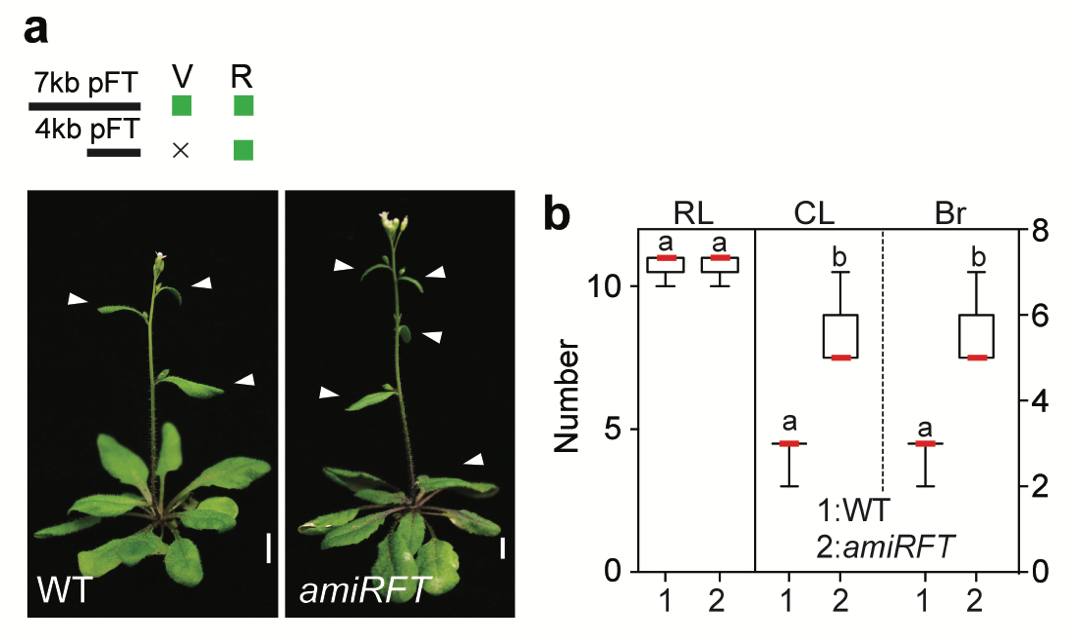
**

**Supplementary Figure 7. Downregulation of *FT* specifically during reproductive development.**

**a, b,** Representative images of the phenotypes of *pFT4kb:amiRFT* plants, in which an artificial microRNA specific to *FT ^11^* was expressed from a minimal *FT* promoter active only during the reproductive phase ^12^. V: vegetative phase, R: reproductive phase (a). Plants were grown in long-day photoperiod. *pFT4kb:amiRFT* plants did not delay onset of reproduction. Plants formed the same number of rosette leaves (RL) as the wild type (b). However, the switch to flower formation occurred significantly later. More cauline leaves (CL) and branches (Br) formed relative to the wild type (b). Quantification, n = 15 independent plants. Box plot-median (red line), upper and lower quartiles (box edges), and minima and maxima (whiskers). Letters: significantly different groups *p*-value < 0.05 based on Kruskal-Wallis test with Dunn's *post hoc* test. Scale bars, 1 cm.

**
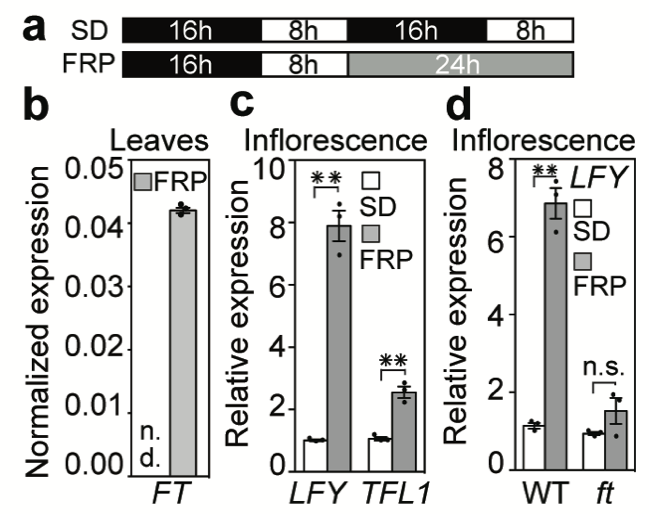
**

**Supplementary Figure 8. FT promotes *LFY* accumulation.**

**a**, Diagram for photoperiod triggered upregulation of *FT* in 42-day-old short-day-grown plants. **b - d**, Change in gene expression after far-red light enriched photoinduction (FRP). Gene expression was normalized to that of *UBQ10*. *FT* expression is strongly elevated in fully expanded true leaves (b) which results in FT protein accumulation at the shoot apex ^13^. (c) FRP caused strong upregulation of *LFY* expression in inflorescences. *TFL1* is moderately upregulated consistent with the known increase in *TFL1* levels at the onset of the reproductive phase ^14^. (d) No significant *LFY* upregulation by FRP was observed in the *ft-10* null mutant (see also **Supplementary Fig.12a**). (b - d) Expression is normalized over that of *UBQ10.* (c, d) Relative gene expression compared to wild-type SD grown plants. Shown are mean ± SEM of three independent biological experiments (black dots). *p*-values: unpaired one-tailed *t*-test ** *LFY* = 0.002; ** *TFL1* = 0.008 (c) ** *LFY* = 0.002, n.s. = 0.11 (d). n.d.: not detectable. The normalized expression values were: *LFY* in SD (0.0024±0.0001), after FRP (0.0185±0.0012); *TFL1* in SD (0.0293±0.0016), after FRP (0.0703±0.0051) for (c) and *LFY* in SD WT (0.0025±0.0002), after FRP WT (0.0152±0.0015); *LFY* in SD *ft* (0.0019±0.0001), after FRP *ft* (0.0031±0.0012) for (d).


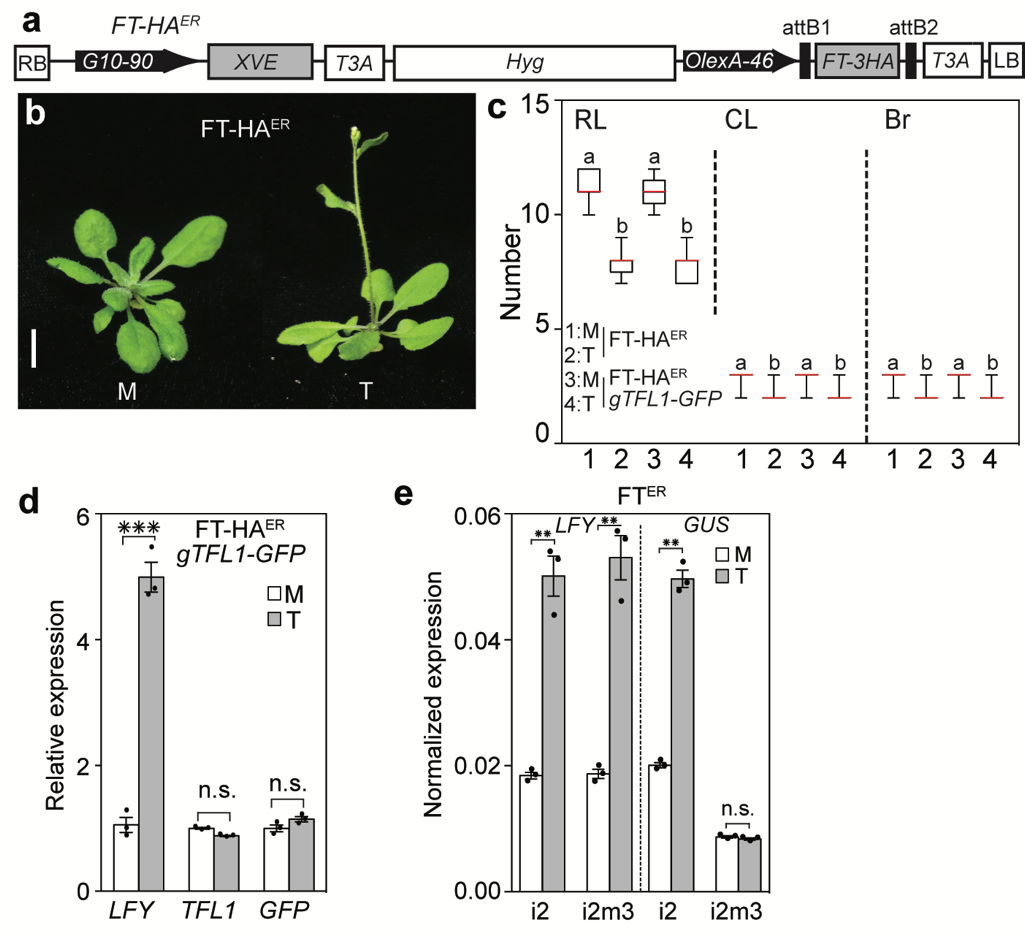


**Supplementary Figure 9. Steroid activated FT causes precocious onset reproductive development and of flower formation.**

**a**, FT-HA^ER^ construct, see Supplementary Figure 5a for details. **b**, Early flowering phenotypes of plants with inducible increased FT. Plants were treated with 10 μmol beta-estradiol (T) or mock (M) solution from day 5 of growth onward every other day until bolting. Scale bars: 1 cm. **c**, Quantification of phenotypes in (b). RL: rosette leaf number, CL: cauline leaf number, Br: branch number. M: mock, T: steroid treatment. Box plot-median (red line; n = 15 plants), upper and lower quartiles (box edges), and minima and maxima (whiskers). Letters above boxes indicate significantly different groups *p*-value < 0.05 based on Kruskal-Wallis test with Dunn's *post hoc* test. **d**, FT-HA^ER^ activation leads to increased *LFY* accumulation in 12-day-old long-day grown plants, while endogenous *TFL1* or transgene *gTFL1*-*GFP* levels do not change. Gene expression was normalized to *UBQ10* and is displayed relative to the mock (M) condition for better comparison. Normalized values (mean±SEM) were: *LFY* (0.0025±0.0003 (M), 0.0118±0.0006(T)); *TFL1* (0.0150±0.0006 (M), 0.0136±0.0002 (T)), and GFP (0.0059±0.0003 (M), 0.0068 ±0.0002 (T)). **e**, Effect of FT-HA^ER^ induction by steroid treatment (4 hrs) in 12-day-old long-day-grown plants on endogenous *LFY* (left) or on a *LFY* reporter (right). Both wild type (pLFYi2:GUS) and bZIP binding site mutated (pLFYi2m3:GUS) reporters were assayed. Expression was normalized over that of *UBQ10.* (d, e) Shown are mean ± SEM of three independent biological experiments (black dots). *p*-value, unpaired one-tailed *t*-test: *** *LFY* = 0.003, n.s. *TFL1*= 0.09, n.s. *GFP* = 0.05 (d); ** *LFY* in pLFYi2 = 0.0051 or ** in pLFYi2m3 = 0.0053, ** *GUS* in pLFYi2 = 0.0011 or n.s. in pLFYi2m3 = 0.13 (e).


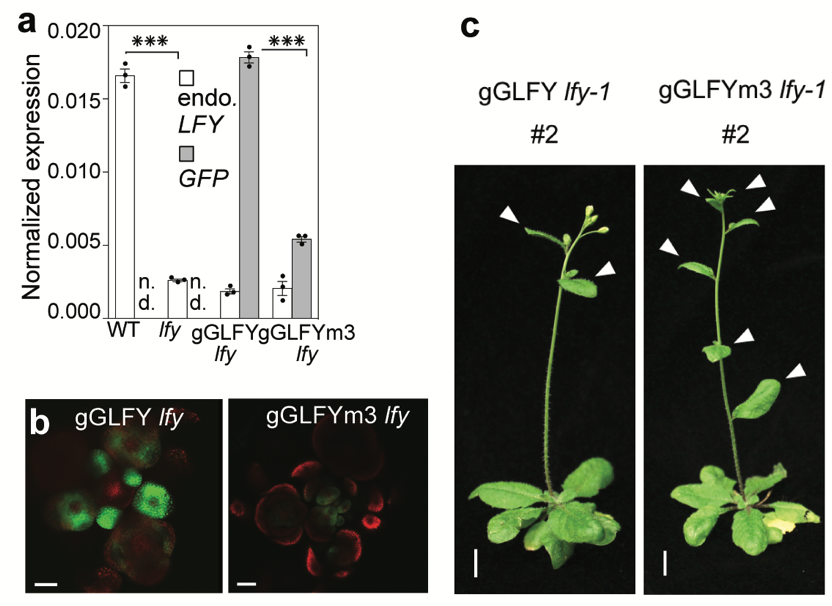


**Supplementary Figure 10. bZIP binding site mutations strongly reduce *LFY* accumulation.**

**a, b,** *LFY* expression in long-day-grown gGLFY *lfy-1* and gGLFYm3 *lfy-1* plants on the basis of qRT-PCR (a) or confocal imaging (b). (a) Expression of *GFP* (grey) and endogenous *LFY* (white) was normalized over that of *UBQ10.* Shown are mean ± SEM of three independent biological experiments (black dots). *p*-value unpaired one-tailed *t*-test. *** endo. *LFY* (wt/*lfy*) = 0.0005, *** *GFP* (gGLFY *lfy*/gGLFYm3 lfy GFP) = 3E-05. n.d.: not detectable. (b) Top view of the inflorescence visualizing GFP signal of gGLFY (left) and gGLFYm3 (right). The image for gGLFYm3 was taken at higher gain (1.3x) and laser power (2.7x) than that of gGLFY. Scale bars, 2 mm. **c,** Rescue of *lfy-1* null mutants by genomic GFP-tagged LFY or a bZIP binding site mutated version thereof (gGLFYm3) under long-day conditions. Arrowheads indicate branches formed on the main stem. Scale bars, 1 cm.


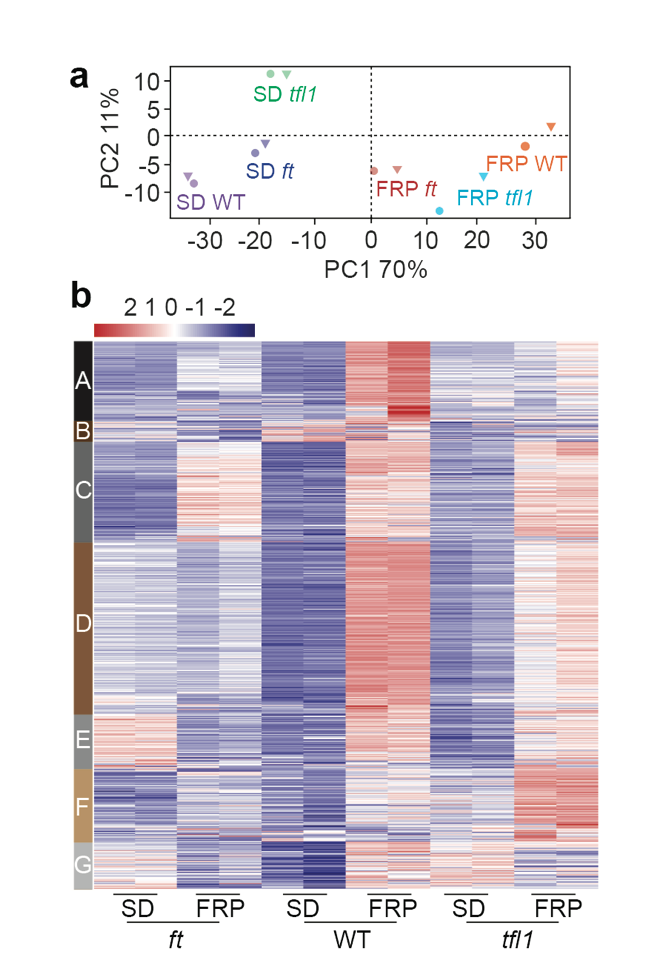


**Supplementary Figure 11. RNA-seq with and without a single far-red enriched photoperiod induction in short-day grown plants.**

**a**, Principle Component Analysis of normalized reads of dissected inflorescences from *ft-10,* wild-type or *tfl1-1* plants. Plants were grown for 42-days in short-day photoperiod ± photoperiod induction. **b**, K-means clustering of all significantly (adjusted p-value cut-off ≤ 0.005) de-repressed genes. DESeq2 normalized expression values are shown for each replicate. Only cluster C lacks FT dependence.


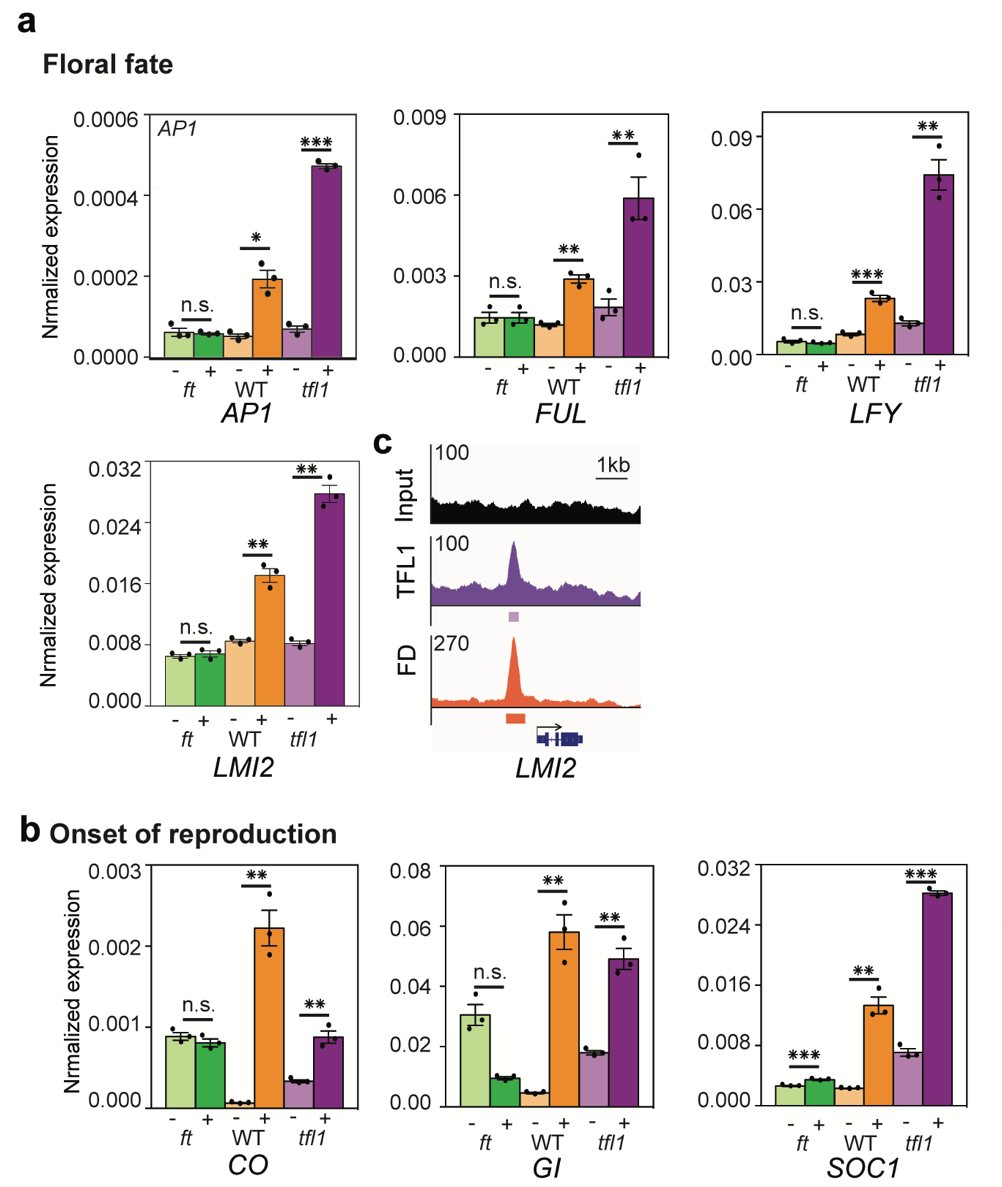


**Supplementary Figure 12. Validation of FT-dependence for TFL1 and FD bound genes involved in onset of reproductive development and flower fate.**

**a, b,** Independent biological replicates of 42-day-old short-day grown plants treated with (+) or without (-) a single far-red enriched photoperiod (FRP). qRT-PCR confirms FT-dependent de-repression of TFL1-FD complex targets linked to onset of flower fate (a) or onset of reproduction (b). SOC1 alone is significantly de-repressed upon FRP treatment in *ft* mutants. Expression is normalized over that of *UBQ10.* Shown are mean ± SEM of three independent biological experiments (black dots). (a, b) *p*-value, unpaired one-tailed *t*-test. From left to right *AP1*: n.s. = 0.65 , * = 0.011, *** = 1E-06; *FUL:* n.s. = 0.5, ** = 0.004, ** = 0.008; *LFY*: n.s. = 0.85, *** = 0.0008, ** = 0.005; *LMI2*: n.s. = 0.28, ** = 0.005, ** = 0.001; *CO*: n.s. = 0.84, ** = 0.005, ** = 0.009; *GI*: n.s. = 0.98, ** = 0.005, ** = 0.006; *SOC1*: *** = 0.0004, ** = 0.005, *** = 2E-05. c. Browser screenshot of *LMI2*, which promotes onset of flower formation together with *LFY* ^15^. Significant peaks (MACS2 summit q value ≤ 10^-10^) are marked by horizontal bars, with the color saturation proportional to the -log 10 q value (as for the narrowPeak file format in ENCODE).


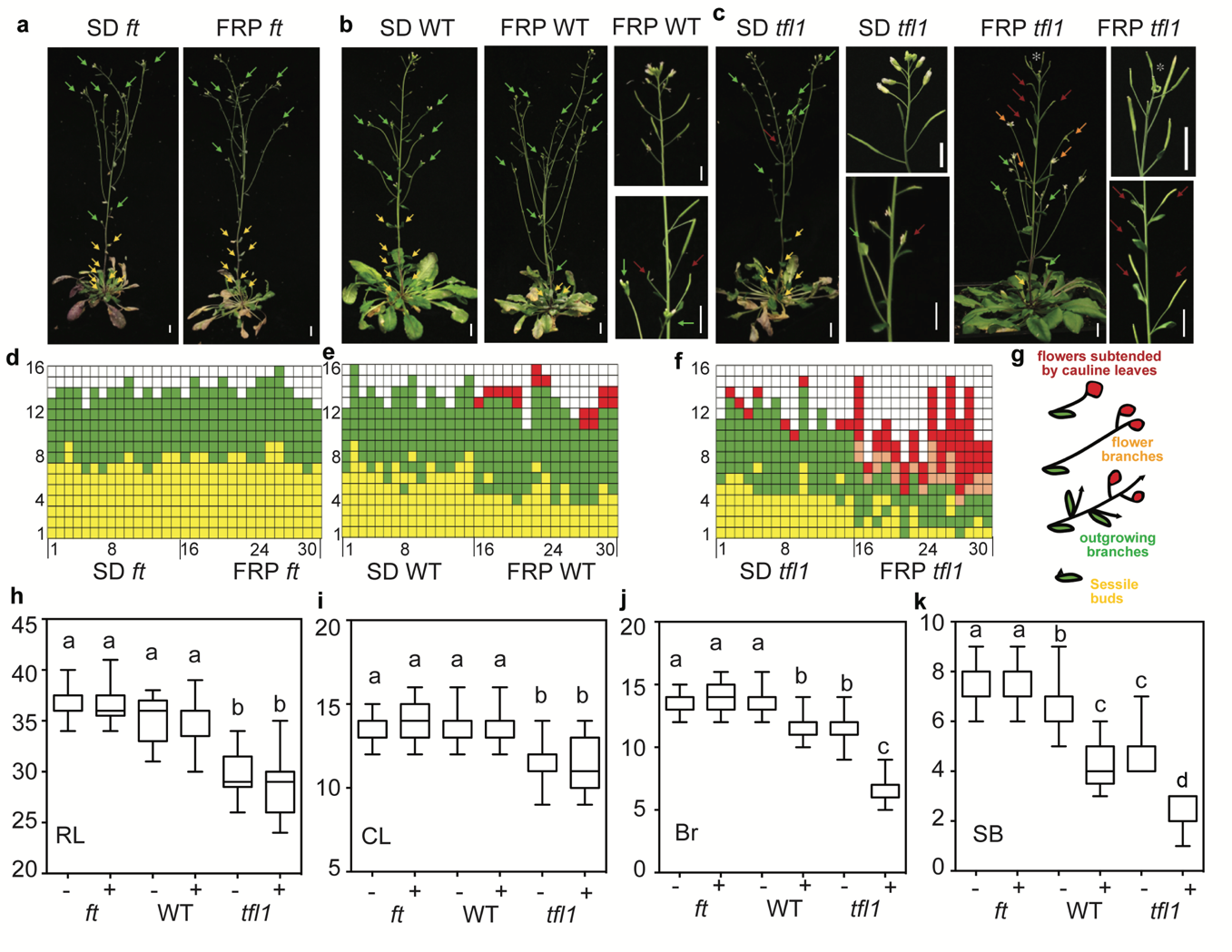


**Supplementary Figure 13.** **Effect of a single far-red light enriched photoperiod (FRP) on *ft-10* mutants, wild type and *tfl1-1*.**

**a - c**, Representative plant images. Yellow arrows: sessile buds. Green arrows: outgrowing branches. Orange arrows: flower branches (a single or two flowers borne at the end of a branch- like petiole subtended by a cauline leaf). Red arrows: flowers subtended by cauline leaves. Scoring was terminated when ‘regular’ flowers not subtended by cauline leaves formed. Asterisk (*) indicates a terminal flower. Scale bar = 1 cm. **d - f**, Identity and fate of primordia formed on the inflorescence from the bottom (node 1) to the top (node 16). Colour coding is as in (a-c): Yellow: sessile buds. Green: outgrowing branches. Orange: flower branches. Red: flowers subtended by cauline leaves. **g,** Schematic of the types of structures formed and colour key for (a - f). **h** - **k**, Phenotype quantification. Box plot-median (red line; n = 15 plants), upper and lower quartiles (box edges), and minima and maxima (whiskers). RL, rosette leaves (h), CL: cauline leaves (i), Br, branches (j), SB: sessile buds (k). -, no FRP treatment; +, a single FRP treatment. Letters above boxes indicate significantly different groups (*p*-value < 0.05) based on Kruskal-Wallis test with Dunn's *post hoc* test. Compared to untreated plants, a single FRP triggered formation of significantly fewer branches in *tfl1* mutants and in the wild type, indicating a switch to floral fate. As the number of cauline leaves, which subtend branches but not flowers in *Arabidopsis*, did not changed, we propose that axillary branch meristems switched to flower fate (in accordance with Ref ^16^). In addition, FRP triggered significant branch outgrowth in *tfl1* mutants and the wild type relative to untreated plants (fewer sessile buds formed). Onset of the reproductive phase was unchanged in these plants. This is expected as FRP was applied at day 42, after the plants had terminated vegetative development. No significant effect of FRP treatment was detectable in the *ft* mutant.


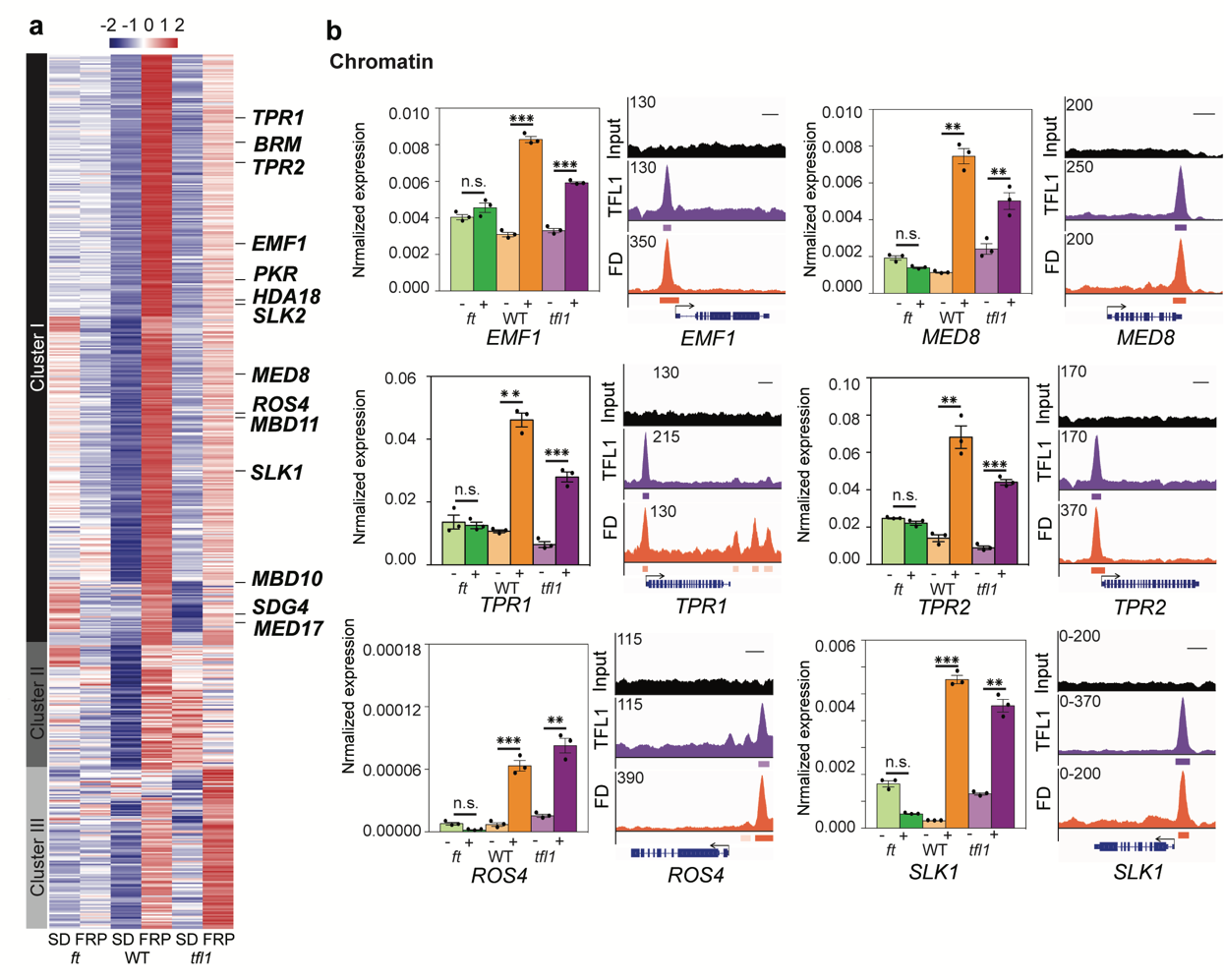


**Supplementary Figure 14. Chromatin regulators bound by TFL1 and FD and de-repressed by FRP.**

**a**, Chromatin regulators and transcriptional co-regulators targets of TFL1-FD complex belong to k-means cluster I. **b,** Left: qRT-PCR of independent biological replicates confirms FT-dependent de-repression of genes directly repressed by the TFL1-FD complex. Plants were treated with (+) or without (-) a single far-red enriched photoperiod (FRP). Expression is normalized over that of *UBQ10.* Shown are mean ± SEM of three independent experiments (black dots). *p*-values based on unpaired one-tailed *t*-test. *EMF1*: n.s. = 0.09, *** = 7E-06, *** = 0.0001; *MED8:* n.s. = 0.98, ** = 0.002, ** = 0.008; *TPR1*: n.s. = 0.65, ** = 0.001, *** = 0.0006; *TPR2:* n.s. = 0.92, ** = 0.006, *** = 0.0001; *ROS4*: n.s. = 0.97, *** = 0.0009, ** = 0.005; *SLK1*: n.s. = 0.99, *** = 0.0004, ** = 0.002. Right: Browser screenshots for input, TFL1 and FD ChIP-seq. Significant peaks (MACS2 summit q val ≤ 10^-10^) are marked by horizontal bars, with the color saturation proportional to the -log 10 q value (as for the narrowPeak file format in ENCODE). Direct TFL1-FD targets include mediator complex subunits (*MED*), which link to Pol II transcription ^17^, co-repressor complexes linked to histone de-acetylation such as SEUSS-LIKE (*SLK*) and TOPLESS-RELATED (*TPR*) ^18^ and HISTONE DEACETYLASE 18 (*HDA18*). BRAHMA (*BRM*) is a SWI/SNF chromatin remodeler ^18^, REPRESSOR OF SILENCING 4 (*ROS4*) a histone acetyltransferase ^19^, EMBRYONIC FLOWER 1 (*EMF1*) and *PICKLE RELATED 1* (*PKR*) are linked to Polycomb repression ^20^, ASH1-RELATED 3/SET DOMAIN GROUP 4 (*AHR3/SDG4*) is a histone methyltransferase ^21^, METHYL-CPG-BINDING DOMAIN 10 and 11 (*MBD10/11*) are putative methyl-DNA binding proteins ^22^.

**Supplementary Table 1.** Gene Ontology terms enriched in genes with TFL1 and FD binding peaks.

| **Description** | **FDR** |
| --- | --- |
| response to abiotic stimulus | 4.50E-35 |
| response to stimulus | 1.30E-30 |
| response to endogenous stimulus | 1.40E-28 |
| regulation of gene expression | 5.20E-22 |
| developmental process | 2.60E-14 |
| anatomical structure development | 3.20E-13 |
| response to stress | 1.90E-12 |
| photosynthesis | 1.40E-10 |
| post-embryonic development | 3.50E-10 |
| cell communication | 9.90E-10 |
| signal transduction | 2.70E-09 |
| cellular biosynthetic process | 1.30E-08 |
| reproductive structure development | 4.70E-08 |
| generation of precursor metabolites and energy | 1.50E-06 |
| flower development | 5.90E-06 |

Significant Peaks (MACS2 q value ≤ 10^-10^) were annotated to genes as described in the Materials and Methods. GO slim was implemented in AgriGO v2.0 (Ref. ^23^) and GO terms associated with Yekutieli (FDR under dependency) significance level < 10^-5^ were identified.

**Supplementary Table 2.** Primers used

| Primer name | Sequence (5' to 3') | Note |  |
| --- | --- | --- | --- |
| Cloning |  | pENTR-DTOPO |  |
| F1 | caccAACAGCAGCAGAGACGGAGAAAGAA | LFY exon 2 |  |
| R1 | TCGTACAAGTGGAACAGATAATC |  |  |
| F2 | caccGGATCCATTTTTCGCAAAGGAAAGT | pLFY |  |
| R2 | AATCTATTTTTCTCTCTCTCTCTATCACTCTCTT |  |  |
| R3 | CTGTCCAATCATCTACATATAAATTGTCACAATC | pLFYi1 |  |
| R4 | CTATTTACACATTTTCCCCAAC | pLFYi1i2 |  |
| GUS-FW | caccATGTTACGTCCTGTAGAAACC | GUS |  |
| GUS-RV | TCATTGTTTGCCTCCCTGCT |  | |
| FT-FW | caccATGTCTATAAATATAAGAGACC | FT-3HA |  |
| FT-3HA-RV | CTAAGCGTAATCTGGAACGTCATATGGATAGGATCCT GCATAGTCCGGGACGTCATAGGGATAGCCCGCATAG TCAGGAACATCGTATGGGTAAAAGATGTTAATTAACCC AAGTCTTCTTCCTCCGCAGCCAC |  |  |
| FT-FV | CTAAAGTCTTCTTCCTCCG | FT |  |
| TFL1-FW | caccATGGAGAATATGGGAACTA | TFL1 |  |
| TFL1-RV | CTAGCGTTTGCGTGCAGCG |  |  |
| FD-FV | caccATGTTGTCATCAGCTAAGCATCA | FD |  |
| FD-RV | TCAAAATGGAGCTGTGGAAGACC |  |  |
|  |  | pENTR3C |  |
| gLFY-FW | cagtcgactggatccggtacGGATCCATTTTTCGCAAAGG | gLFY |  |
| gLFY-RV | ggtctagatatctcgagtgcCTAGAAACGCAAGTCGTCGCC |  |  |
| GLFY-FW | cggttccacctccgctgcagATGAGTAAAGGAGAAGAACTTTTCACT | gGLFY |  |
| GLFY-RV | tgcggtgtcaccggctgttgCTGCAGCTGTTTGTATAGTTCATC |  |  |
| gLFY-FW | cagtcgactggatccggtacGGATCCATTTTTCGCAAAGG | gLFY-GUS |  |
| gLFY-RV2 | GAAACGCAAGTCGTCGCC |  |  |
| LFYGUS-FW | gcggcgacgacttgcgtttcATGTTACGTCCTGTAGAAACC |  |  |
| LFYGUS-RV | ggtctagatatctcgagtgcTCATTGTTTGCCTCCCTGCT |  |  |
| pFT4-FW1 | gtcgactggatccggtaccgCAAGCTTTTGTTGGACATTC | pFT4:amiRFT |  |
| pFT4-RV1 | CTTTGATCTTGAACAAACAGG |  |  |
| amiRFT-FW | acctgtttgttcaagatcaaagCTGCAAGGCGATTAAGTTGGGTAAC |  |  |
| amiRFT-RW | atatctcgagtgcggccgcgaGCGGATAACAATTTCACACAGGAAACAG |  |  |
| 1-amiR-ft-2 | gaTTGGTTATAAAGGAAGAGGCCtctctcttttgtattcc | amiRFT |  |
| 2-amiR-ft-2 | gaGGCCTCTTCCTTTATAACCAAtcaaagagaatcaatga |  |  |
| 3-amiR-ft-2 | gaGGACTCTTCCTTTTTAACCATtcacaggtcgtgatatg |  |  |
| 4-amiR-ft-2 | gaATGGTTAAAAAGGAAGAGTCCtctacatatatattcct |  |  |
| pRS300-A | CTGCAAGGCGATTAAGTTGGGTAAC |  |  |
| pRS300-B | GCGGATAACAATTTCACACAGGAAACAG |  |  |
| pFT4-FW2 | atcttcgaacacgtgaggccCAAGCTTTTGTTGGACATTC | pFT4:GW |  |
| pFT4-RV2 | cttttttgtacaaacttgtgatcCTTTGATCTTGAACAAACAGG |  |  |
| bZIPm-FW | CCA ATG TTA ACA AGT GTG GAA  ACC GAC GAA GAT GTA AAC GAA GG | FD binding site  mutation |  |
| bZIPm-RV | GGG GAA GTG GCT AGA GGC AAA AAG |  |  |
| Y1HLFYe2-FW | aagcttgaattcgagctcgGGTTATCTGAGGAACCGGTGCAG | pAbAi-LFYe2 pAbAi-LFYe2m3 |  |
| Y1HLFYe2-RV | tacatacagagcacatgccCTTGGTGGGGCATTTTTCGC |  |  |
| ChIP-qPCR |  |  |  |
| P1-FW | TGCCTGCAGGTCGACTCTAAT |  |  |
| P1-RV | CGTCTCTGCTGCTGTTGGTG |  |  |
| P2-FW | GAGACAGAGGGAGCATCCGTT |  |  |
| P2-RV | GGATCCTCTAGATCGAACCACTTTGT |  |  |
| LFY-1-FW | GCGAAGAAAGCAAGAAGAAAG |  |  |
| LFY-1-RV | CTCGGTCAGCCCATTACATT |  |  |
| LFY-2-FW | AGCCAGTATTGCCAACTTTCC |  |  |
| LFY-2-RV | TCTTAAGATACATGGCCAACCT |  |  |
| LFY-3-FW | CCTACGTGTCAAATTATGAATGG |  |  |
| LFY-3-RV | GCATTTATGTGTAGATGTAATGTGATG |  |  |
| LFY-4-FW | ATTGGTTCAAGCACCACCTC |  |  |
| LFY-4-RV | CCCTCTAAACCACCAAGTCG |  |  |
| LFY-5-FW | GAAATATCAAATATCGCACGTTTT |  |  |
| LFY-5-RV | CGGGCATAGAAATGTTGAGAA |  |  |
| LFY-6-FW | GGAGGAAGTGGTTACTGGGA |  |  |
| LFY-6-RV | TGACGTCAGCATTGGTTTCT |  |  |
| LFY-7-FW | GAAGACGTCAACGAAGGTGA |  |  |
| LFY-7-RV | GGATGCTCCCTCTGTCTCTC |  |  |
| LFY-8-FW | GGTTTGGGGACAGAGAGACA |  |  |
| LFY-8-RV | CCAGGCTCCGTTACGATAAA |  |  |
| LFY-9-FW | AGTCTATTGCTCGGCGGATA |  |  |
| LFY-9-RV | TACAAGCAATGGCACAGAGC |  |  |
| LFY-10-FW | AACGCTCATCCTCGTCTCTC |  |  |
| LFY-10-RV | CTCCAAATGGCAAAGCTGAC |  |  |
| TA3-FW | CTGCGTGGAAGTCTGTCAAA |  |  |
| TA3-RV | CTATGCCACAGGGCAGTTTT |  |  |
| GI-FW | AGCCATCCAGACAGAAGTGGA |  |  |
| GI-RV | ACCACTTGTCTCTACTGTACCACA |  |  |
| CO-FW | AAGCTCAACTAGCTGCAAGAGG |  |  |
| CO-RV | TTGATGGTGTGGGAGCTGGT |  |  |
| SOC1-FW | CAAGGTCAACTACGTGGCAT |  |  |
| SOC1-RV | CGTGCGACGTCGAATCAAT |  |  |
| FUL-FW | GCCAATGAGGACTCGACAACA |  |  |
| FUL-RV | GTTGGGTTTCGCGTGAGTCT |  |  |
| AP1-FW | CCAGTGGTCCGTACAATGTTACT |  |  |
| AP1-RV | GCGTGTCGACTTCTCATTGGT |  |  |
| LMI2-FW | TGAAAACTACGGCTTTGACTTGT |  |  |
| LMI2-RV | ACAGAGTCACGAAACAATCCA |  |  |
| qRT-PCR |  |  |  |
| qLFY-FW | AGGTACGCGAAGAAATCAGG |  |  |
| qLFY-RV | CGCTCTTCTGAGAGCATTTG |  |  |
| qTFL1-FW | CCTGCACTGGATCGTTACAA |  |  |
| qTFL1-RV | TGGCAATTCATAGCTCACCA |  |  |
| qFT-FW | TGGCCGCAGTTTTCTACAAT |  |  |
| qFT-RV | CTCATTTTCCTCCCCCTCTC |  |  |
| qGUS-FW | TCTACTTTACTGGCTTTGGTCG |  |  |
| qGUS-RV | CGTAAGGGTAATGCGAGGTAC |  |  |
| qGFP-FW1 | CCATTACCTGTCCACACAATC |  |  |
| qGFP-RV1 | CCCTCTAAACCACCAAGTCG |  |  |
| qGFP-FW2 | GACGACGGCAACTACAAGAC |  |  |
| qGFP-RV2 | GTCCTCCTTGAAGTCGATGC |  |  |
| qAP1-FW | AAAACAGCATGCTTTCTAAACAGA |  |  |
| qAP1-RV | GTGGCCTTGGTTCTGCTG |  |  |
| qFUL-FW | TCGAATATTCCACCGACTCTTGC |  |  |
| qFUL-RV | TTTGTGAAACGTCTCGGCCAAC |  |  |
| qLIM2-FW | AAAGGTCCTTGGACGCCTGAAG |  |  |
| qLIM2-RV | TCTTCCCACAGCGAAGTAAACCAG |  |  |
| qLOG5-FW | TAGCAGCAGCGGAAAGAGAGAG |  |  |
| qLOG5-RV | TCAATCTCCTCGTCACCAGCTC |  |  |
| qTPPJ-FW | TCTACACCAGGAGCCAAAGTGG |  |  |
| qTPPJ-RV | AGCTCGCTCCATTTCTTCTCGTC |  |  |
| qTPPH-FW | GAGACTAGCGCGTCTTATTCACTG |  |  |
| qTPPH-RV | ACGTTGCAAGAACTCCATAACCTC |  |  |
| qBIM1-FW | AACCGTCGAAGCTCTGTCGTTC |  |  |
| qBIM1-RV | TGGGTGGACGGTTGAATGACTTG |  |  |
| qSMXL6-FW | GGGAATAACAGGCAGCAGTAAGTG |  |  |
| qSMXL6-RV | TTCTGTAGAGCAGCGGTTTGCG |  |  |
| qAFP2-FW | AACGGGAGGAGGTAGTTCATCGAG |  |  |
| qAFP2-RV | TGCAGCTGTTTGATGATCCTTGC |  |  |
| qMP-FW | GCTCGGGTTGGAAGCTTGTATATG |  |  |
| qMP-RV | TTACGCATCCCACAAACTCTTCC |  |  |
| qROS4-FW | GGCGTGCTTATCAATGCAGGTG |  |  |
| qROS4-RV | CTCGCTGCATATCCAACATGTGC |  |  |
| qEMF1-FW | GTGGGAGGGATTTGTGCAGTTC |  |  |
| qEMF1-RV | CATCTGTTAATCCCTCTGCCTCAG |  |  |
| qMED8-FW | GCAGCAGCAACTACTTGCACAAC |  |  |
| qMED8-RV | GCATTTGATGTTGCCCATGTGACG |  |  |
| qSLK1-FW | CACCTCGTGCAAAGCAGAGATTG |  |  |
| qSLK1-RV | AAGATCACACTGCCACATATCCG |  |  |
| qUBQ10-FW | ATGGGTCCTTCAGAGAGTCCT |  |  |
| qUBQ10-RV | CTTGGTCCTAAAGGCCACCT |  |  |
| Genotyping |  |  |  |
| lfy-FW | ATTGGTTCAAGCACCACCTC |  |  |
| lfy-RV | AATCGTCTCCGTTCAGCTCT |  |  |
| Note: FW, forward; RV, reverse | |  |  |

**Supplementary Table 3.** Summary of plant lines generated.

| Construct | Transformed into | Crossed to |
| --- | --- | --- |
| *gTFL1-GFP* | *tfl1-1* |  |
| *TFL1^ER^* | wild type |  |
| *FT-HA^ER^* | wild type | *pLFYi2:GUS*  and *pLFYi2m3:GUS* |
| *FT-HA^ER^* | gTFL1-GFP *tfl1-1* |  |
| *pLFY:GUS* | wild type |  |
| *pLFYi1:GUS* | wild type |  |
| *pLFYi2:GUS* | wild type |  |
| *pLFYi2m3:GUS* | wild type |  |
| *e2* | wild type  gTFL1-GFP *tfl1-1*  *FT-HA^ER^* |  |
| *e2m3* | wild type  gTFL1-GFP *tfl1-1*  *FT-HA^ER^* |  |
| *pFT4kb:amiRFT* | wild type |  |
| *gGLFY* | *lfy-1/+** |  |
| *gGLFYm3* | *lfy-1/+** |  |
| *gLFY:GUS* | wild type |  |
| *gLFYm3:GUS* | wild type |  |

* Heterozygous *lfy-1 -*/+ seed stock was generated by crossing *lfy-1* to line C2723 (Ref. ^24^), which contains two fluorescent markers flanking the *lfy-1* locus (*NAP:eGFP NAP:dsRED*, C2723)*.* Homozygous *lfy-1* plants were identified in the T2 generation by fluorescence marker selection and confirmed by genotyping.

**Supplementary References**

1. Wu, X. *et al.* Modes of intercellular transcription factor movement in the Arabidopsis apex. *Development* **130**, 3735-3745 (2003).

2. Heinz, S. *et al.* Simple Combinations of Lineage-Determining Transcription Factors Prime cis-Regulatory Elements Required for Macrophage and B Cell Identities. *Molecular Cell* **38**, 576-589 (2010).

3. Collani, S., Neumann, M., Yant, L. & Schmid, M. FT Modulates Genome-Wide DNA-Binding of the bZIP Transcription Factor FD. *Plant Physiol* **180**, 367-380 (2019).

4. Goretti, D. *et al.* TERMINAL FLOWER1 Functions as a Mobile Transcriptional Cofactor in the Shoot Apical Meristem. *Plant Physiol* **182**, 2081-2095 (2020).

5. Jin, R. *et al.* LEAFY is a pioneer transcription factor and licenses cell reprogramming to floral fate. (2020).

6. Sayou, C. *et al.* A SAM oligomerization domain shapes the genomic binding landscape of the LEAFY transcription factor. *Nature Communications* **7**, 11222 (2016).

7. Curtis, M.D. & Grossniklaus, U. A Gateway Cloning Vector Set for High-Throughput Functional Analysis of Genes in Planta. *Plant Physiology* **133**, 462-469 (2003).

8. Blazquez, M.A., Soowal, L.N., Lee, I. & Weigel, D. LEAFY expression and flower initiation in Arabidopsis. *Development* **124**, 3835-3844 (1997).

9. Taoka, K. *et al.* 14-3-3 proteins act as intracellular receptors for rice Hd3a florigen. *Nature* **476**, 332-5 (2011).

10. Kaneko-Suzuki, M. *et al.* TFL1-Like Proteins in Rice Antagonize Rice FT-Like Protein in Inflorescence Development by Competition for Complex Formation with 14-3-3 and FD. *Plant Cell Physiol* **59**, 458-468 (2018).

11. Schwab, R., Ossowski, S., Riester, M., Warthmann, N. & Weigel, D. Highly Specific Gene Silencing by Artificial MicroRNAs in Arabidopsis. *The Plant Cell* **18**, 1121-1133 (2006).

12. Adrian, J. *et al.* cis-Regulatory Elements and Chromatin State Coordinately Control Temporal and Spatial Expression of FLOWERING LOCUS T in Arabidopsis. *The Plant Cell* **22**, 1425-1440 (2010).

13. Andrés, F. & Coupland, G. The genetic basis of flowering responses to seasonal cues. *Nature Reviews Genetics* **13**, 627 (2012).

14. Conti, L. & Bradley, D. TERMINAL FLOWER1 is a mobile signal controlling Arabidopsis architecture. *Plant Cell* **19**, 767-78 (2007).

15. Pastore, J.J. *et al.* LATE MERISTEM IDENTITY2 acts together with LEAFY to activate <em>APETALA1</em>. *Development* **138**, 3189-3198 (2011).

16. Hempel, F.D., Zambryski, P.C. & Feldman, L.J. Photoinduction of flower identity in vegetatively biased primordia. *Plant Cell* **10**, 1663-76 (1998).

17. Larivière, L. *et al.* Structure of the Mediator head module. *Nature* **492**, 448-451 (2012).

18. Martin-Arevalillo, R. *et al.* Structure of the *Arabidopsis* TOPLESS corepressor provides insight into the evolution of transcriptional repression. *Proceedings of the National Academy of Sciences* **114**, 8107 (2017).

19. Qian, W. *et al.* A Histone Acetyltransferase Regulates Active DNA Demethylation in *Arabidopsis*. *Science* **336**, 1445 (2012).

20. Calonje, M., Sanchez, R., Chen, L. & Sung, Z.R. EMBRYONIC FLOWER1 Participates in Polycomb Group-Mediated *AG* Gene Silencing in *Arabidopsis*. *The Plant Cell* **20**, 277 (2008).

21. Kumpf, R. *et al.* The ASH1-RELATED3 SET-Domain Protein Controls Cell Division Competence of the Meristem and the Quiescent Center of the Arabidopsis Primary Root. *Plant Physiology* **166**, 632-643 (2014).

22. Berg, A. *et al.* Ten members of the Arabidopsis gene family encoding methyl‐CpG‐binding domain proteins are transcriptionally active and at least one, AtMBD11, is crucial for normal development. *Nucleic Acids Research* **31**, 5291-5304 (2003).

23. Yan, H. *et al.* agriGO v2.0: a GO analysis toolkit for the agricultural community, 2017 update. *Nucleic Acids Research* **45**, W122-W129 (2017).

24. Wu, G., Rossidivito, G., Hu, T., Berlyand, Y. & Poethig, R.S. Traffic Lines: New Tools for Genetic Analysis in Arabidopsis thaliana. *Genetics* **200**, 35-45 (2015).
