# Supplementary material for: TERMINAL FLOWER 1-FD complex target genes and competition with FLOWERING LOCUS T
Source: Nat Commun. 2020 Oct 12;11:5118. doi: 10.1038/s41467-020-18782-1 (PMC7550357; doi:10.1038/s41467-020-18782-1)
Supplement: Supplementary file 3 — Descriptions of Additional Supplementary Files [file 41467_2020_18782_MOESM3_ESM.pdf]

Descriptions of Additional Supplementary Files

Supplementary Data 1 TFL1-FD bound and regulated genes
