# Supplementary material for: TERMINAL FLOWER 1-FD complex target genes and competition with FLOWERING LOCUS T
Source: Nat Commun. 2020 Oct 12;11:5118. doi: 10.1038/s41467-020-18782-1 (PMC7550357; doi:10.1038/s41467-020-18782-1)
Supplement: Supplementary file 5 — Reporting Summary [file 41467_2020_18782_MOESM5_ESM.pdf]

nature research

Corresponding author(s): Doris Wagner

Last updated by author(s): Aug 18, 2020

Reporting Summary

Nature Research wishes to improve the reproducibility of the work that we publish. This form provides structure for consistency and transparency in reporting. For further information on Nature Research policies, see Authors & Referees and the Editorial Policy Checklist .

Statistics

For all statistical analyses, confirm that the following items are present in the figure legend, table legend, main text, or Methods section.

n/a	Confirmed	
☐	☒	The exact sample size (*n*) for each experimental group/condition, given as a discrete number and unit of measurement
☐	☒	A statement on whether measurements were taken from distinct samples or whether the same sample was measured repeatedly
☐	☒	The statistical test(s) used AND whether they are one- or two-sided<br>*Only common tests should be described solely by name; describe more complex techniques in the Methods section.*
☒	☐	A description of all covariates tested
☐	☒	A description of any assumptions or corrections, such as tests of normality and adjustment for multiple comparisons
☐	☒	A full description of the statistical parameters including central tendency (e.g. means) or other basic estimates (e.g. regression coefficient) AND variation (e.g. standard deviation) or associated estimates of uncertainty (e.g. confidence intervals)
☐	☒	For null hypothesis testing, the test statistic (e.g. *F*, *t*, *r*) with confidence intervals, effect sizes, degrees of freedom and *P* value noted<br>*Give P values as exact values whenever suitable.*
☒	☐	For Bayesian analysis, information on the choice of priors and Markov chain Monte Carlo settings
☒	☐	For hierarchical and complex designs, identification of the appropriate level for tests and full reporting of outcomes
☐	☒	Estimates of effect sizes (e.g. Cohen's *d*, Pearson's *r*), indicating how they were calculated

Our web collection on statistics for biologists contains articles on many of the points above.

Software and code

Policy information about availability of computer code

Data collection	All software used (including the version and parameter, when not default) is indicated in the Methods section. A githhub link to code is provided.
Data analysis	All software used (including the version and parameter, when not default) is indicated in the Methods section. A githhub link to code is provided.

For manuscripts utilizing custom algorithms or software that are central to the research but not yet described in published literature, software must be made available to editors/reviewers. We strongly encourage code deposition in a community repository (e.g. GitHub). See the Nature Research guidelines for submitting code & software for further information.

Data

Policy information about availability of data

All manuscripts must include a data availability statement. This statement should provide the following information, where applicable:

- Accession codes, unique identifiers, or web links for publicly available datasets
- A list of figures that have associated raw data
- A description of any restrictions on data availability

Accession code for genomic datasets is provided

Field-specific reporting

Please select the one below that is the best fit for your research. If you are not sure, read the appropriate sections before making your selection.

☒ Life sciences ☐ Behavioural & social sciences ☐ Ecological, evolutionary & environmental sciences

Life sciences study design

All studies must disclose on these points even when the disclosure is negative.

Sample size	The sample size was chosen on the basis of prior studies that showed significant effects with similar samples sizes (for example see Winter et al., Developmental Cell 2011, Xiao et al., Nature Genetics 2017 and Yamaguchi N. et al., Science 2015, Chung et al., NC 2020)
Data exclusions	No data was excluded from the analysis
Replication	All attempts at replication were sucessfull
Randomization	Sample allocation into groups was random
Blinding	The investigators were not blinded

Reporting for specific materials, systems and methods

We require information from authors about some types of materials, experimental systems and methods used in many studies. Here, indicate whether each material, system or method listed is relevant to your study. If you are not sure if a list item applies to your research, read the appropriate section before selecting a response.

Materials & experimental systems

n/a	Involved in the study	
☐	☒	Antibodies
☒	☐	Eukaryotic cell lines
☒	☐	Palaeontology
☒	☐	Animals and other organisms
☒	☐	Human research participants
☒	☐	Clinical data

Methods

n/a	Involved in the study	
☐	☒	ChIP-seq
☒	☐	Flow cytometry
☒	☐	MRI-based neuroimaging

Antibodies

Antibodies used	anti-GFP antibody (Thermo Fisher Scientific, A-11122; 1:200 dilution)<br>anti-GUS antibody (Abcam, ab50148; 1:200 dilution)
Validation	The antibodies were validated by the suppliers.

ChIP-seq

Data deposition

☒ Confirm that both raw and final processed data have been deposited in a public database such as GEO.

☒ Confirm that you have deposited or provided access to graph files (e.g. BED files) for the called peaks.

Data access links<br>*May remain private before publication.*	*For "Initial submission" or "Revised version" documents, provide reviewer access links. For your "Final submission" document, provide a link to the deposited data.*
Files in database submission	Deposition at GEO: GSE141894<br>ChIPseq<br>FD ChIP: 2 replicates<br>TFL1ChIP : 4 replicates<br>TFL1 ChIP in fd mutant: 2 replicates<br>Control ChIP (no antigen) wild type: 2 replicates<br>Control ChIP (no antigen) fd mutant: 2 replicates<br>Input : 4 replicates in total<br><br>RNA-seq<br>ft mutant no treatment: 3 replicates<br>ft mutant FRP photoperiod shift: 3 replicates<br>tfl1 mutant no treatment: 3 replicates<br>tfl1 mutant FRP photoperiod shift: 3 replicates

wild type no treatment: 3 replicates
wild type FRP photoperiod shift: 3 replicates

Genome browser session (e.g. UCSC)

Provide a link to an anonymized genome browser session for "Initial submission" and "Revised version" documents only, to enable peer review. Write "no longer applicable" for "Final submission" documents.

Methodology

Replicates

Replicates agreed well, data provided in the supplement

Sequencing depth

Experiment	Total number of reads	Reads with MAPQ>=30	Length of reads	Paired or single end
gFD-GUS Rep1 ChIP-Seq	25,695,641	9,212,549	75	SINGLE
gFD-GUS Rep2 ChIP-Seq	26,131,970	10,185,838	75	SINGLE
gTFL1-GFP A Rep1 ChIP-Seq	21,669,805	10,636,053	75	SINGLE
gTFL1-GFP A Rep2 ChIP-Seq	20,580,773	10,067,141	75	SINGLE
gTFL1-GFP B Rep1 ChIP-Seq	22,388,892	11,719,076	75	SINGLE
gTFL1-GFP B Rep2 ChIP-Seq	19,983,625	11,284,465	75	SINGLE
fd_gTFL1-GFP Rep1 ChIP-Seq	20,743,880	8,775,382	75	SINGLE
fd_gTFL1-GFP Rep2 ChIP-Seq	27,505,136	12,369,123	75	SINGLE
Control Rep1 ChIP-Seq	21,687,867	3,946,947	75	SINGLE
Control Rep2 ChIP-Seq	23,161,566	4,647,368	75	SINGLE
Control for fd_gTFL1-GFP R1	30,542,333	8,402,421	75	SINGLE
Control for fd_gTFL1-GFP R2	35,808,196	9,997,036	75	SINGLE
TFL1 Input Rep1 ChIP-Seq	40,751,281	26,558,556	75	SINGLE
TFL1 Input Rep2 ChIP-Seq	40,570,788	27,277,901	75	SINGLE
TFL1B input ChIP-Seq	48,142,717	16,067,082	75	SINGLE
Control input ChIP-Seq	44,701,929	15,510,704	75	SINGLE

Antibodies

anti-GFP antibody (Thermo Fisher Scientific, A-11122; 1:200 dilution)
anti-GUS antibody (Abcam, ab50148; 1:200 dilution)
Antibodies were validated by the supplier and by control ChIP reactions using plants lacking the antigene.

Peak calling parameters

Peak calling is described in the Methods

Data quality

Peak attributes are described in the Methods

Software

Software used is described in the Methods
